# Highly Efficient Solid-State Near-infrared Organic Light-Emitting Diodes incorporating A-D-A Dyes based on *α,β*-unsubstituted “BODIPY” Moieties

**DOI:** 10.1038/s41598-017-01785-2

**Published:** 2017-05-09

**Authors:** Andrea Zampetti, Alessandro Minotto, Benedetta Maria Squeo, Vasilis G. Gregoriou, Sybille Allard, Ullrich Scherf, Christos L. Chochos, Franco Cacialli

**Affiliations:** 1 Department Physics and Astronomy and London Centre for Nanotechnology, University College London, London, WC1H 0AH UK; 2 Advent Technologies SA Patras Science Park, Patra, 26504 Greece; 3 Macromolecular Chemistry Department and Institut for Polymer Technology, Bergische Universität Wuppertal, Wuppertal, Germany

## Abstract

We take advantage of a recent breakthrough in the synthesis of *α,β*-unfunctionalised 4,4-difluoro-4-bora-3a,4a-diaza-s-indacene (BODIPY) moieties, which we symmetrically conjugate with oligothienyls in an unexpectedly stable form, and produce a “metal-free” A-D-A (acceptor-donor-acceptor) oligomer emitting in the near-infrared (NIR) thanks to delocalisation of the BODIPY low-lying lowest unoccupied molecular orbital (LUMO) over the oligothienyl moieties, as confirmed by density functional theory (DFT). We are able to retain a PL efficiency of 20% in the solid state (vs. 30% in dilute solutions) by incorporating such a dye in a wider gap polyfluorene matrix and demonstrate organic light-emitting diodes (OLEDs) emitting at 720 nm. We achieve external quantum efficiencies (EQEs) up to 1.1%, the highest value achieved so far by a “metal-free” NIR-OLED not intentionally benefitting from triplet-triplet annihilation. Our work demonstrates for the first time the promise of A-D-A type dyes for NIR OLEDs applications thereby paving the way for further optimisation.

## Introduction

Organic semiconductors absorbing or emitting in the near-infrared (NIR) range of the electromagnetic spectrum are currently emerging as an unusual class of materials with useful optoelectronic properties. Their most appealing features are solution processing, low-cost of fabrication, and the possibility of preparation on top of flexible, conformable or even stretchable substrates for organic electronic devices, such as organic (OLEDs) and polymer light-emitting diodes (PLEDs)^1–3^. These advantages have commanded attention from both the academic and the industrial sector for their potential in medical (e.g. for photodynamic therapy, pulsi-oxymetry, intracellular imaging), telecommunication (e.g. optical communication), and defence areas (e.g. for night-vision devices, capillary mapping in identification systems, etc.)^4, 5^. In general, materials being explored as NIR absorbers or emitters include organic semiconductors, metal–organic complexes, inorganic nanoparticles and hybrid organic–inorganic perovskite-like compounds that have been exploited as NIR emitters in sandwich type LEDs (mostly with the help of organic charge injection layers)^6–14^. Although the quantum yield in this region of the spectrum is significantly lower than for materials emitting in the visible, owing to the so-called “energy-gap law”, external quantum efficiencies (EQEs) spanning from ~9% to ~24% and over have been achieved in OLEDs incorporating phosphorescent Pt-based porphyrins and carefully-designed derivatives^15–19^. While this is still significantly better than what is achievable with fluorescent NIR materials, the scope and applicability of phosphorescent OLEDs generates significant concerns both because of the use of heavy metals, and the so-called “efficiency roll-off”, that is limiting the efficiency as the current is increased, and that is due to a combination of triplet-triplet annihilation, triplet-polaron quenching, and phosphorescent sites saturation (in various ratios also depending on current density)^18^.

Among the different “metal-free” organic materials investigated so far, the state-of-the-art results in the red/NIR region are obtained by combining electron-donating (or donor, D) and electron deficient (or acceptor, A) building blocks with the most common being “D-A-D” dyes^20^. Triphenylamine (TPA), ethylenedioxythiophene (EDOT), pyrrole, thiophene, and bridged bithiophenes derivatives are commonly-used electron-donating units, whereas electron-deficient moieties such as benzobisthiadiazole, thiadiazoloquinoxaline, diketopyrrolopyrrole, thienoisoindigo and cyclopentadithiophenone moieties have been largely employed as acceptor units^21–29^. Interestingly, Yao *et al*. reported electroluminescence (EL) EQEs >1% from both D-A-D and A-D-A compounds^30, 31^. Such systems benefit from a favourable conformational arrangement of the constituting units, which controls the orbital mixing and leads to the formation of a hybrid localised/charge-transfer (CT) excited state^31^. It was proposed that the localised nature of these states brings about a high radiative rate, whereas the weakly-bound CT nature leads to the formation of a high fraction (>25%) of singlet excitons.

More generally, however, whereas the properties of dyes of the D-A-D type have been studied extensively, different alternation patterns of the D and A moieties and in particular A-D-A dyes have rarely been explored up to now, mainly because of synthetic difficulties associated to the monofunctionalization of the A building block.

**Figure 1:**
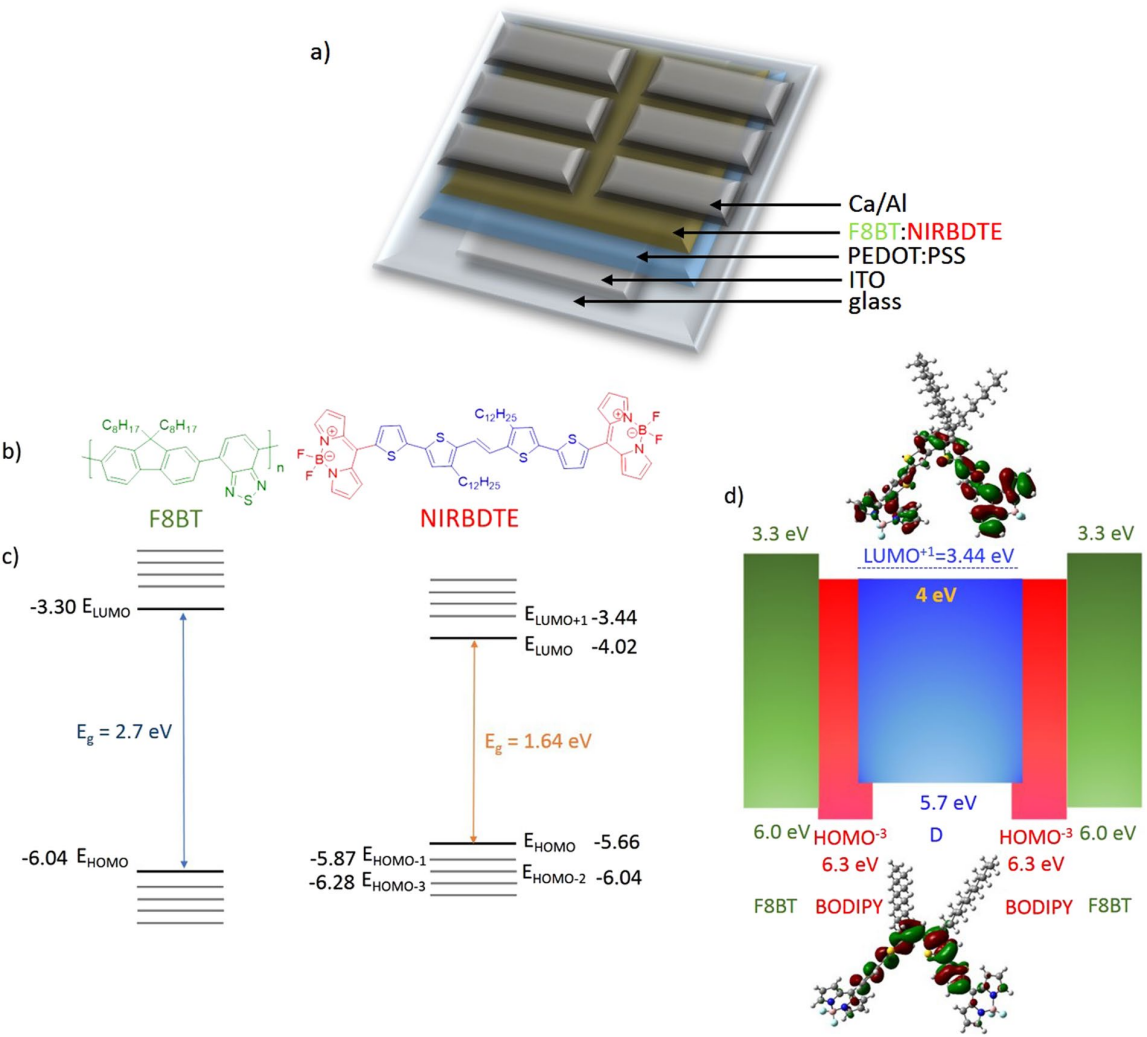
(**a**) Device structure, including details of the different layers, (**b**) chemical structure of F8BT and NIRBDTE, (**c**) the energy levels of F8BT and NIRBDTE and (**d**) the related band diagram (both extracted from the cyclic voltammetry data) of the emitting blend F8BT:NIRBDTE as active layer (in their isolated conditions, before heterostructure formation) with the density-functional theory (DFT) HOMO and LUMO wavefunction plots of NIRBDTE (calculated with the B3LYP/6-31 G(d,p) basis set). The effective HOMO and LUMO of NIRBDTE are those of the part indicated in blue, but we also illustrate in red the local electronic structure of the BODIPY moieties to emphasise the insight gained from DFT calculations of the frontier levels charge distribution, and the “hole-funnelling” effects towards the central bithienyl.

Here, we address this knowledge gap and present a successful approach to the design and synthesis of a novel A-D-A type NIR dye (referred to as NIRBDTE, for brevity, see Fig. 1 for chemical structure) which is then used as the emitter in NIR-OLEDs with the highest efficiency reported so far for a metal-free fluorescent materials emitting at 720 nm. More specifically, inspired by the structural motif of the state-of-the-art D-A-D dye*, 2,3-*bis(4-(diphenylamino)-[1,1-biphenyl]-4-yl) fumaronitrile (TPATCN), we attach two bithienyl moieties on either side of an ethylene linkage to constitute the electron-donating segment. To these bithienyls we then further attach two (terminal) *α,β*-unsubstituted 4,4-difluoro-4-bora-3a,4a-diaza-s-indacene (BODIPY) as the electron deficient (A) blocks. The BODIPYs are connected to the *α,ω*-oligothiophene cores through their meso-positions to achieve linear and symmetrical molecular architectures (affording extended π-conjugation throughout the dye) as well as optimized molecular energetics (*vide infra*). In particular, we decided to anchor two dodecyl side chains on the thiophene rings to obtain sufficient solubility in common organic solvents. Furthermore, with a view to establishing general protocols for (organic) semiconductors design, we consider that BODIPY might be an ideal acceptor end-unit because it has strong electron-withdrawing characteristics, and it is expected to provide both negative inductive (-I) effects originating from “through-bond” polarizations^30^, and so-called mesomeric (-M) effects, originating from π-bond polarizations^32, 33^. Such an approach is expected to facilitate both delocalization and stabilization of charge carriers (e.g. electrons). BODIPY-type dyes are an emerging class of red/NIR emitters (and absorbers)^34–39^, because even if the monomeric BODIPY is a green-emitting fluorophore (quantum efficiency up to 100% with narrow absorption and emission bands <50 nm)^40, 41^, it can be functionalized by means of few synthetic steps so as to shift the energy gap into the NIR^34–36^. In practice, the development of BODIPY-based organic semiconductors and especially those of the *α,β*-unsubstituted forms has so far lagged behind that of other π-deficient units, mainly because of stability issues during the synthesis. However, thanks to a recently developed synthetic protocol, stable *α,β*-unsubstituted BODIPYs functionalized solely on the meso position can be successfully produced and integrated into more complex structures^35^.

## NIRBDTE

First of all, we combined density functional theory (DFT) calculations and cyclic voltammetry (reported in the Supporting Information Figs S6 and S8, respectively) to investigate position and spatial distribution of the frontier energy levels (highest occupied molecular orbital, HOMO, and lowest unoccupied molecular orbital LUMO) that are eventually responsible for the electronic properties of NIRBDTE. DFT calculations were carried out with the ground state geometry optimized at the B3LYP/6-31 G(d,p). The distributions of the frontier molecular orbitals in the ground state are shown in Fig. 1d. From this we immediately notice that the LUMO charge density is well distributed along the entire molecule and does not localize at the two BODIPY acceptor units, as it might have been expected in view of the electron-withdrawing nature of the latter. On the contrary, charge distribution within the HOMO is localized on the thiophenes of the central segment. The computed HOMO and LUMO levels are found at −5.56 eV and −3.06 eV (where the minus sign indicates that the levels are relative to the vacuum energy), respectively, in excellent agreement with atmospheric pressure photoelectron spectroscopy (ionization potential, IP ~ −5.48 eV; Fig. S7 in the electronic supplementary information section, ESI) and cyclic voltammetry (Fig. S8 in the ESI) from which we derive the energy structure reported on the right of Fig. 1c, and further illustrated, also with a view to device applications, in Fig. 1d. Here we also include poly(9,9′-dioctylfluorene-alt-benzothiadiazole) (F8BT), that we later used as the host matrix of our light-emitting diodes (LEDs). Consistently with the DFT results discussed above, the LUMO is set by the BODIPYs, and extends over the full NIRBDTE. This is not the case for the HOMO, whose dominant feature is instead the negligible charge distribution over the BODIPYs. Whereas the NIRBDTE HOMO is thus set by that of the bithienyl at ~5.7 eV below vacuum, the relevant local energy levels on the BODIPY moieties remain those of the parent molecule at ~−6.3 eV. Furthermore, from the DFT calculations we note that introduction of the didodecyl side chains close to the ethylene bond, leads to a “pseudo” 2D conformation of the NIRBDTE, with segments of the latter lying both in-plane and out-of-plane due to cis-geometric isomerism of the ethylene bond (see also ﻿Fig. S6 in ﻿the ESI).

Although it is well known that DFT calculations with the B3LYP/6-31 G(d,p) basis set tend to over-planarise the molecules and over-delocalise the wavefunctions^42^, the localisation of the HOMO on the BODIPY moieties in our case is an indication that for this particular molecule such over-delocalisation effects do not appear to be particularly severe, even if “*a priori*” we cannot completely rule out a mild effect on the LUMOs. Interestingly, consideration of the experimental luminescence lifetime (Fig. S9 in the ESI, for emission from a state largely localized on the thiophene-rich moiety) brings further support to the scenario above, in which the LUMO is essentially delocalised. In fact, if this were not the case, and the LUMO of the thiophene-rich unit were higher-lying (e.g. at the position of the LUMO^+1^ ~ −3.44 eV, or higher) we would expect a detectable charge-transfer character for this state, which would result in a longer lifetime than experimentally observed (~1.2 ns).

Importantly, we consider that such an intramolecular energy structure should favour a “hole funnelling” effect towards the central part of the molecule in the presence of a substantially homogeneous distribution of electrons on a relatively low-lying LUMO, that could for example be populated effectively from a host polymer (such as F8BT) in a guest-host emitting layer. The added potential bonus of this structure is that the resulting excitons should therefore tendentially localize on the central part of the molecule, and thus be less available to quenching by neighbouring moieties or contaminants.

In Fig. 1a we display the schematic structure of our LEDs, in which, as mentioned above, we used F8BT as the host matrix to enable formation of a ‘so-called’ type I or straddling heterojunction (HOMO_F8BT_ ~ −6.0 eV and LUMO_F8BT_ ~ −3.3 eV), i.e. such that the bandgap of one semiconductor is completely contained in the bandgap of the other one (alternatively such that the differences of the energy levels between the two organic semiconductors, ΔHOMO and ΔLUMO, have opposite signs). Such a straddling heterojunction favours the exciton localization in the oligomer and makes exciton dissociation unlikely, contrary to what happens in “staggered” or “Type II” heterojunctions (i.e. with ΔHOMO and ΔLUMO having the same sign) with large frontier energies mismatches. The scheme of type I and II heterojunctions are also reported in the ESI (Fig. S10). We incorporate different concentrations (0.5%, 1%, 5% and 10% wt) of NIRBDTE in blends with F8BT, in conventional solution-processed PLEDs with ITO/PEDOT:PSS anodes and Ca/Al cathodes to investigate the influence of the NIR oligomer on the device operation. Atomic force microscopy, AFM, imaging, which we report in Fig. S11 in the ESI, does not show formation of large structures suggestive of significant phase separation between the components (only minor features are observed on the 10 nm scale or so, but present also for the pure F8BT films, and therefore not attributable to phase separation between NIRBDTE and F8BT).

## Results and Discussion

**Figure 2:**
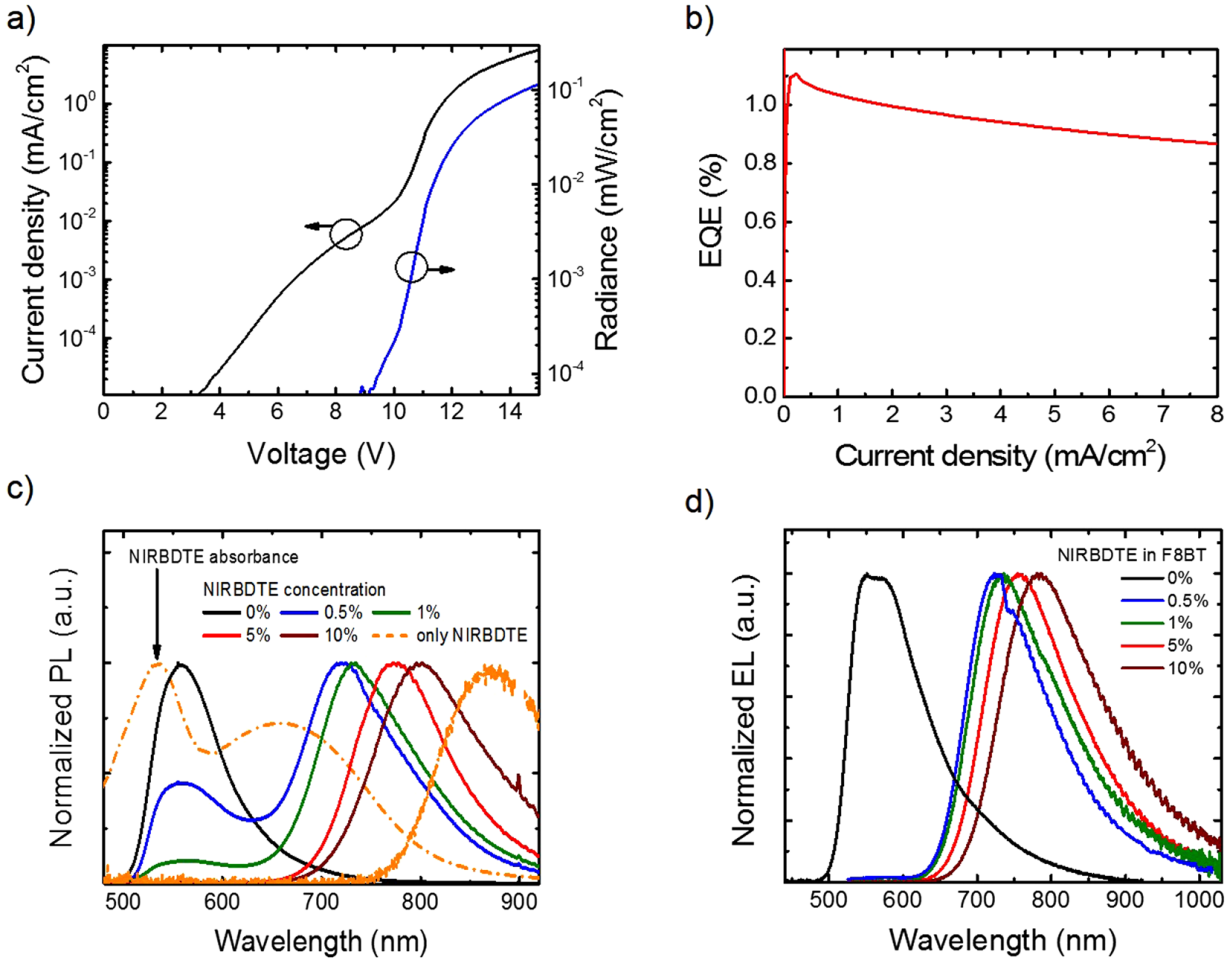
(**a**) J-V-R curves and (**b**) EQE of PLED incorporating the blend of F8BT with the NIRBDTE at 0.5% wt. (**c**) PL spectra (excited at 450 nm) of the solid films incorporating different NIRBDTE concentration in the host F8BT and the EL spectra of PLEDs incorporating these films as emissive layers.

In Fig. 2a–d we report the current density-voltage-radiance (J-V-R) characteristics, the external quantum efficiency (EQE) of diodes incorporating the 0.5% wt NIRBDTE -F8BT blend, and the PL and EL spectra of solid films with different NIRBDTE loadings. J-V-R and EQE curves of the highest efficient PLEDs incorporating the blend of F8BT with NIRBDTE at 1%, 5% and 10% wt are reported in the Supporting Information (Fig. S12). Remarkably, the LEDs with 0.5% NIRBDTE loading exhibited EQEs up to 1.1% (Fig. 2b) with a turn-on voltage (V_ON_) of 9.43 ± 0.05 V and an EL emission (~65% in NIR region) of ~0.11 mW/cm^2^ peaked at 720 nm (Fig. 2a,d). Emission from F8BT is quenched in the presence of NIRBDTE due to efficient energy transfer, consistently with the good spectral overlap between the F8BT PL (Fig. 2c, black solid line) and the absorbance of NIRBDTE (orange dash-dotted line). The ~1.1% EQE in the solid state was measured at 0.22 mA/cm^2^ with a driving voltage of 11 V (Fig. 2a) and represents the state-of-the-art for fluorescent “metal-free” emitting materials, in which only singlets fluorescence can be harvested, such that the maximum achievable internal quantum efficiency is limited to 1/4 of the PL efficiency (*Φ*_*PL*_), consistently with simple spin-statistics arguments. To put this number in an appropriate context, we further calculate the theoretical maximum EQE that can be obtained from devices of this kind, by taking into account the measured PL efficiency of our active layers, i.e. *Φ*_*PL*_ = 20 ± 2% (F8BT incorporating 0.5% wt NIRBDTE, measured on spectrosil substrates) and by considering the surface out-coupling efficiency *ξ* ^43, 44^. The latter can be estimated for example by using the published values of the active layer’s refractive index (~1.75 for F8BT above 700 nm, having disregarded the perturbation of NIRBDTE in this case, given the slight concentration), so that we can further calculate *ξ* to vary from 0.16 to 0.24, respectively, in the case of isotropic or in-plane dipoles. In the ideal case of unit charge injection efficiency (*γ*) ~1, we thus expect an “ideal” EQE to vary from 0.56% to 1.47% (also taking into account the propagation of the error on *Φ*_*PL*_ measurements) thereby confirming the excellent quality of our devices, and the limited margins for improvements in terms of device architecture. In fact, further improvements can be envisaged by improving *Φ*_*PL*_, or by moving to a phosphorescent material, as reported in the literature with EQEs now surpassing 24%^19^, but this is outside our scope, which we intentionally limited to fluorescence to avoid heavy/toxic metals.

As reported in Fig. 2c, at increasing NIRBDTE concentrations, the NIR emission component gains intensity with respect to the F8BT residual band, and progressively red-shifts (from 720 nm to 800 nm). Furthermore, the PL efficiency decreases by increasing the NIRBDTE content in F8BT from 19% (at 1% wt) to 5% (at 10% wt). Such reduction is entirely expected as a result of both the reduction of the energy gap, and because of the aggregation-induced quenching of the oligomer blended with F8BT. PL decay measurements are also reported in the ESI (Fig. S9). These show a ~1 ns single exponential decay, which confirms the dominant singlet nature of the radiative exciton. As reported in Fig. 2d, EL spectra also exhibit a red-shift of the NIRBDTE emission with concentration, however and contrary to what observed in the PL spectra, there is no residual F8BT contribution at 0.5% wt and 1% wt. We assign this effect to preferential formation of excited species directly onto the guest chromophores, which are also expected to mediate transport (energy selective in its nature, and occurring preferentially through low-energy sites).

**Figure 3:**
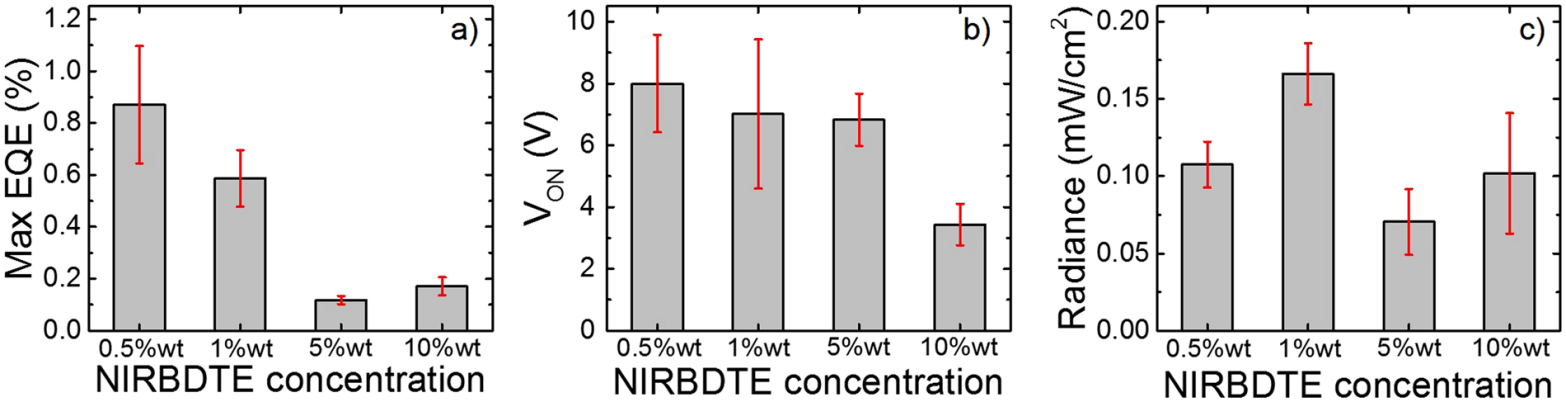
Mean and the related standard deviation (in red) of (**a**) the maximum external quantum efficiency (EQEs), (**b**) the turn-on voltage (V_ON_) and (**c**) the radiance of PLEDs incorporating different concentration of NIRBDTE (0.5%, 1%, 5% and 10% wt) in blend with F8BT as host material for a set of 8 devices. V_ON_ is the voltage at which the emission is ~10 times the noise level and the radiance is the light intensity (mW/cm^2^) at the saturation of the emission.

In Fig. 3a–c we compare the OLEDs key parameters (EQE, V_ON_ and radiance) as a function of the NIRBDTE loading. From Fig. 3a, in particular, we note that the devices with 0.5% loading feature the maximum EQE (1.1%), and that this gradually decreases with increasing loading (on average from ~0.9% for the 0.5% NIRBDTE concentration, to ~0.2% at 1% guest by weight). At the same time however, the 0.5% devices also feature the highest turn-on voltage (Fig. 3b −9.45 ± 0.05 V on average). This is significantly higher than for F8BT (~2.5 V) and we attribute it to charge trapping by the dopant in the emissive layer^45^. The progressive decrease of the turn-on voltage with NIRBDTE concentration, taken together with the PL efficiency reduction, and red-shift of the luminescence, strongly supports the interpretation that NIRBDTE molecules are well-dispersed in the host matrix for the 0.5% devices, but increasingly less so as the loading is increased^45^. The radiance from the different devices is compared in Fig. 3c, and ranges from ~0.11 mW/cm^2^ (at ~52 mA/cm^2^ for 10% wt NIRBDTE) to ~0.24 mW/cm^2^ (at ~23 mA/cm^2^ for 1% wt NIRBDTE). Interestingly we find that the emitted radiance is maximised in the 1% NIRBDTE devices (0.19 mW/cm^2^), as a result of the interplay between efficiency and driving conditions, which allows easier charge injection and transport compared to the more efficient 0.5% devices.

## Conclusions

We provide significant insight into the “scarcely-studied” class of purely organic and red/NIR emitting A-D-A type dyes based on *α,β*-unsubstituted BODIPY moieties as A units embedded in a wider gap F8BT as emissive layer for OLEDs. The emitting devices incorporating the BODIPY NIR emitter at a relatively low concentration of 0.5% wt exhibit maximum EQEs up to 1.1%, with an EL emission peaked at 720 nm (~65% in the “conventional” NIR region, i.e. λ > 700 nm). In terms of maximum efficiency these results represent the current state of the art for NIR emitters not taking advantage of heavy/toxic metals to induce phosphorescence, as emission efficiency is expected to decrease with decreasing gaps according to the so called energy-gap law. We propose that our preliminary results demonstrate the potential of the ADA motif, and that can guide further design and optimization of NIR emitters for biomedical, security, and communication applications.

## Methods

The oligomer NIRBDTE (MW ≈ 1000 g/mol) was synthesized by Advent Technologies SA (the synthesis details are reported in the Supporting Information), whereas F8BT (Mw ≈ 255919 g/mol) was purchased from OSSILA. We deposited films of F8BT/NIRBDTE (~90 nm thick) onto clean Spectrosil substrates via spin-coating for the UV-Vis spectrum detection. The devices were built on top of indium-doped tin oxide (ITO) -coated glass (Colorado Concept Coatings LLC, ~20 Ω/square) cleaned in an ultrasonic bath with acetone and isopropyl alcohol (10 min each step) and treated with an oxygen plasma (15 min at 10.2 W)^46–48^. PEDOT:PSS (Sigma-Aldrich) was spin coated on top of the ITO-coated glass substrates in air and thermally treated inside a N_2_ glove-box to obtain 50 nm thick films. All the following steps were carried out inside the glove-box. Both F8BT and BODIPY were dissolved in *toluene* (10 mg/ml). Pristine F8BT and different concentrations (0.5%, 1%, 5% and 10% wt) of NIRBDTE in blend with F8BT were spin coated as emissive layers on top of the anode so as to obtain ~90 nm thick films. The following step consisted in high vacuum thermal evaporation (1·10^−6^ mbar) of a Ca (30 nm thick) cathode with a 150 nm thick Al protective layer. The set-up used for EL characterization of devices and films were reported in the literature^49^. Film thicknesses were measured using a Dektak stylus profilometer. PL spectra of F8BT, 1, 5 and 10% wt blends and NIRBDTE thin films on spectrosil substrates were measured at room temperature with excitation from a 450 nm laser diode module (average power <1 mW, collimated beam) and collected with an ANDOR-Shamrock 163 spectrometer coupled with an ANDOR-Newton charge-coupled device (CCD) unit. The PL efficiency of the thin films was measured by means of an integrating sphere. We excited the samples with the same low-power 450 nm laser diode module mentioned above. Time-resolved PL measurements were carried out with a time-correlated single photon counting (TCSPC) spectrometer previously reported^50^. The AFM images have been acquired with a Bruker Dimension Icon AFM.

## Electronic supplementary material

Supplementary Information
