# Supplementary material for: Highly Efficient Solid-State Near-infrared Organic Light-Emitting Diodes incorporating A-D-A Dyes based on α,β-unsubstituted “BODIPY” Moieties
Source: Sci Rep. 2017 May 9;7:1611. doi: 10.1038/s41598-017-01785-2 (PMC5431651; doi:10.1038/s41598-017-01785-2)
Supplement: Supplementary file 1 — Supplementary Information [file 41598_2017_1785_MOESM1_ESM.doc]

**Supporting information**

**Highly Efficient Solid-State Near-infrared Organic Light-Emitting Diodes incorporating A-D-A Dyes based on α,β-unsubstituted "BODIPY" Moieties**

By *Andrea Zampetti, Alessandro Minotto, Benedetta Maria Squeo, Vasilis G. Gregoriou, Sybille Allard, Ullrich Scherf, Christos L. Chochos* and Franco Cacialli**

**Experimental**

All reactions were treated as air and light sensitive and performed under argon and in the dark. All glassware used were washed using teepol surfactant, rinsing with excess water, acetone and methylene dichloride and dried in an oven at 120 °C. All solvents and reagents were sourced commercially from Aldrich, except (E)-1,2-bis(3-dodecyl-5-(trimethylstannyl)thiophen-2-yl)ethane which was obtained from Solarmer Materials Inc.

**Synthetic Procedures**

**2,2'-((5-octylthiophen-2-yl)methylene)bis(1H-pyrrole) [1]**

5-bromothiophene-2-carbaldehyde (2.13 g, 11.16 mmol) was dissolved in excess of pyrrole (31 mL, 446.4 mmol). The resulting mixture was degassed for 30 min by argon and then 0.1 mL of trifluoroacetic acid were added. The reaction was stirred at room temperature for an hour and the crude product was diluted with methylene chloride and washed three times with sodium hydroxide (NaOH) aq. 0.1 N and dried on magnesium sulphate (MgSO_4_). The solvent was removed under reduced pressure. The product was purified by chromatography on silica gel using hexane/ethyl acetate 9:1 as eluent. The yellow-green solid product obtained with a yield of 95% (3.29 g).

^1^H NMR (CDCl_3_, 400 MHz): δ 5.65 (s, 1Η), 6.07 (m, 2Η), 6.18 (m, 2Η), 6.64(dd, ^3^J= 6,64Hz, 1Η), 6.70 (t, 3J = 6.71Hz, 2Η), 8.0 (s, 2Η).

**Bromo functionalized-BODIPY monomer [2]**

Monomer 1 (3.29 g, 10.70 mmol) and the DDQ (2.43 g, 10.70 mmol) were dissolved in dry toluene (54 mL + 54 mL) and then added in a predegassed three necked flask. After 30 min the predistilled Hünig's base (DIPEA, 8.8 mL) was added and the mixture stirred at room temperature for 90 min. Finally, the boron trifluoride diethyl etherate (9 mL) was added and the mixture was stirred at 80°C for 2h and then cooled at room temperature. The crude product was washed with water and dried on magnesium sulphate (MgSO_4_). The solvent was removed under reduced pressure and the product was purify by silica gel chromatography using a mixture of dichloromethane:hexane 2:1 as eluent. The product obtained as a pink solid with a yield of 49% (1.87 g).

^1^H NMR (CDCl_3_, 400 MHz): δ 6.6(d, 2H), 7.27 (dd, ^3^J=7.27, 3H), 7.35 (d, 1H), 7.96 (s, 2H).

a

a

b

b

c

c

e

d

a

e

c

b

d

**Figure S1**. ^1^H-NMR of Intermediate BODIPY monomer **2**.

**Synthesis of A-D-A targeted dye**

To a mixture of BODIPY intermediate monomer 2 (0.22 g, 0.26 mmol), distannyl-1,2-di(2-thienyl)ethylene (DTE) (0.23 g, 2,5 eq) and tetrakis(triphenylphosphine)palladium (5%, 14.9 mg) was added toluene (5 mL). The mixture was stirred overnight at 110 °C. The crude product was cooled down and toluene was removed under reduced pressure. The crude product was purified by silica-gel column chromatography using a mixture of hexane: dichloromethane 7:3 eluent and then recrystallized from hexane to afford compound NIRBDTE. The product obtained as a purple solid with a yield of 76% (0.21 g).


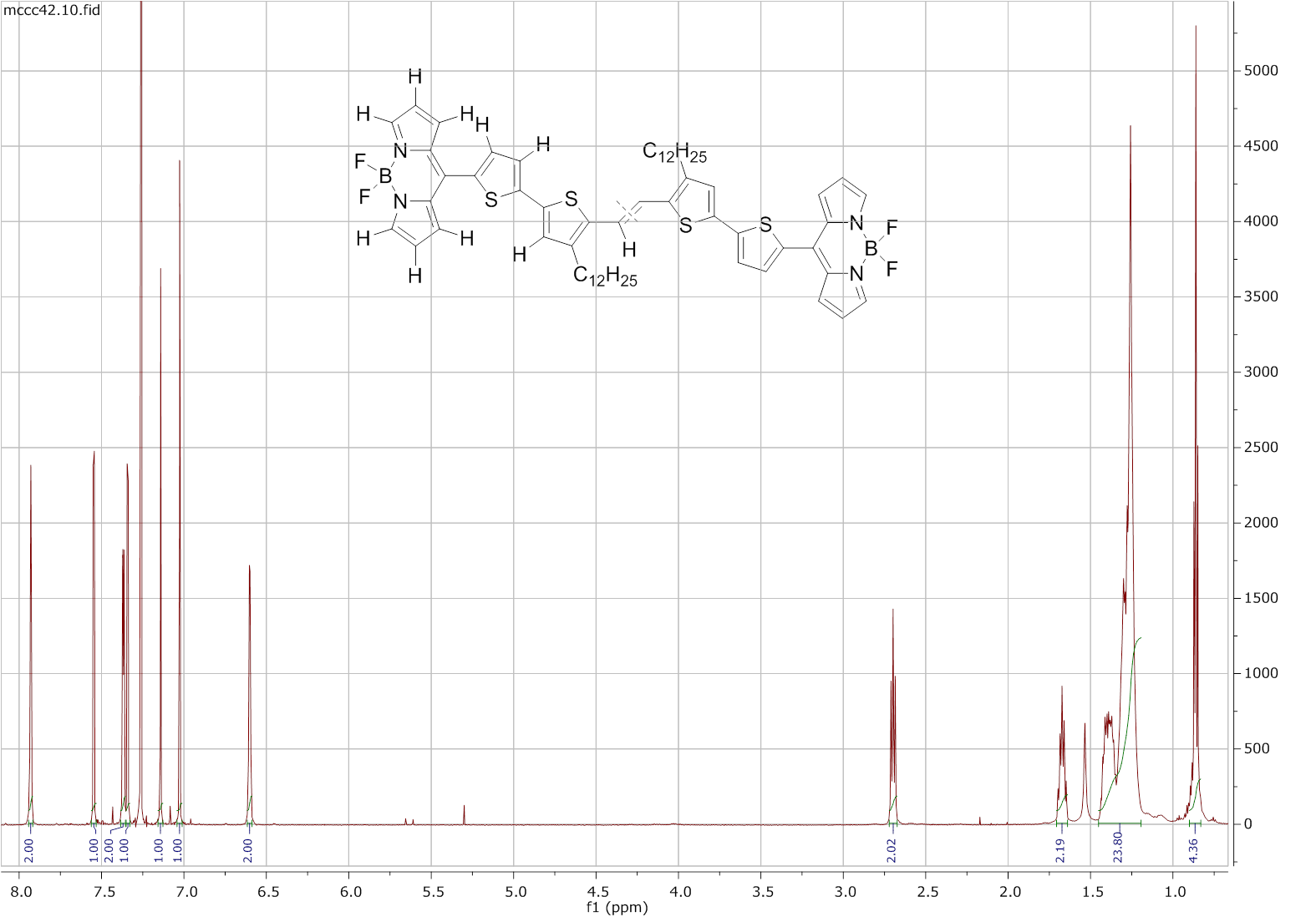
^1^H NMR (CDCl_3_, 400 MHz): δ 1,30(m, 42Η), 1,67 (m, 4Η), 2.70 (t, 4Η), 6,60 (dd, ^3^J = 6.60Hz, 4Η), 7.02 (s, 2Η), 7.14 (s, 2Η), 7.35 (d, 2Η), 7.37 (d, 4Η), 7.55 (d, 2Η), 7.93 (s, 4Η).

**Figure S2**. ^1^H-NMR of NIRBDTE.

**
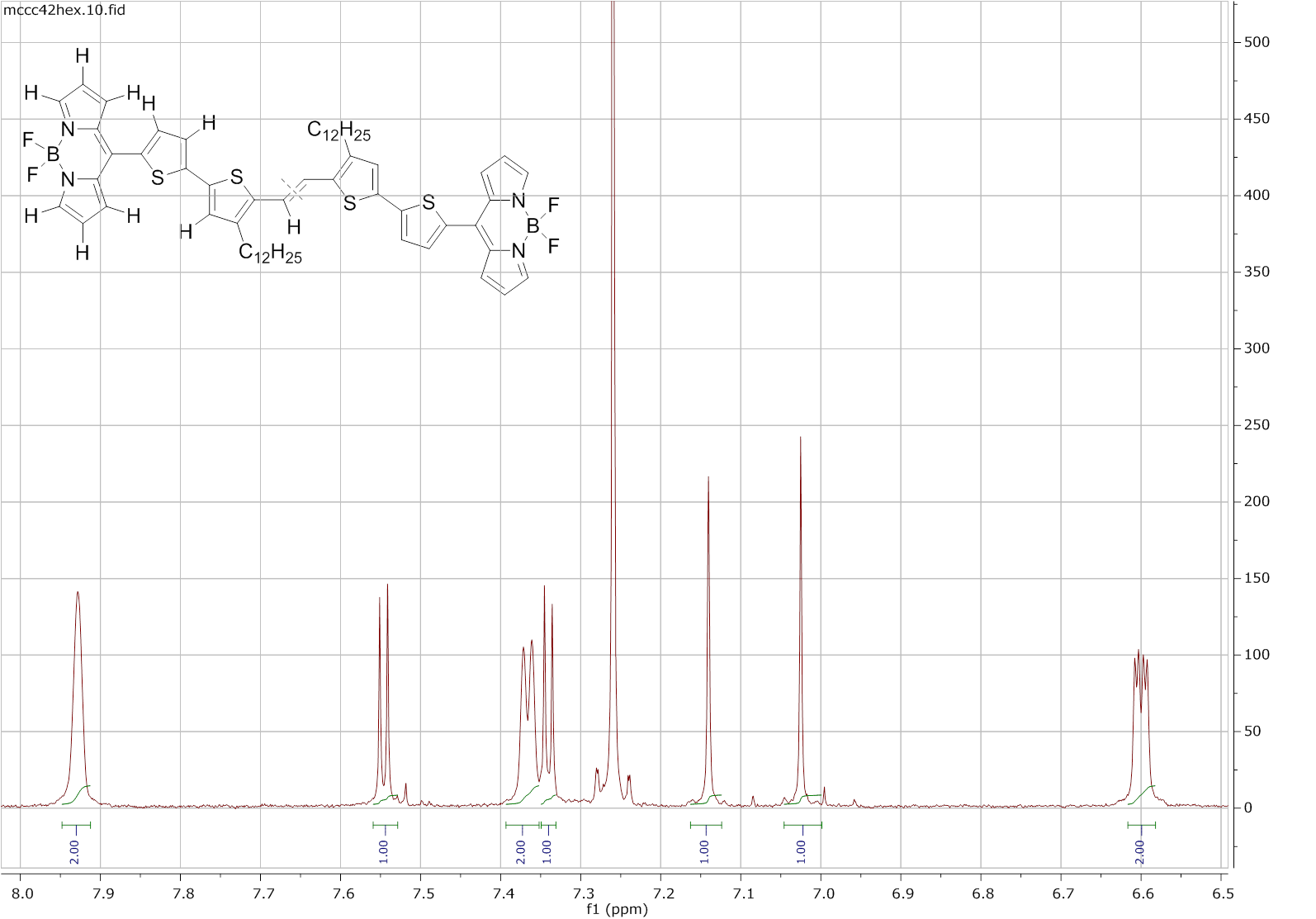
**

**Figure S3**. ^1^H-NMR of NIRBDTE (aromatic region).

**
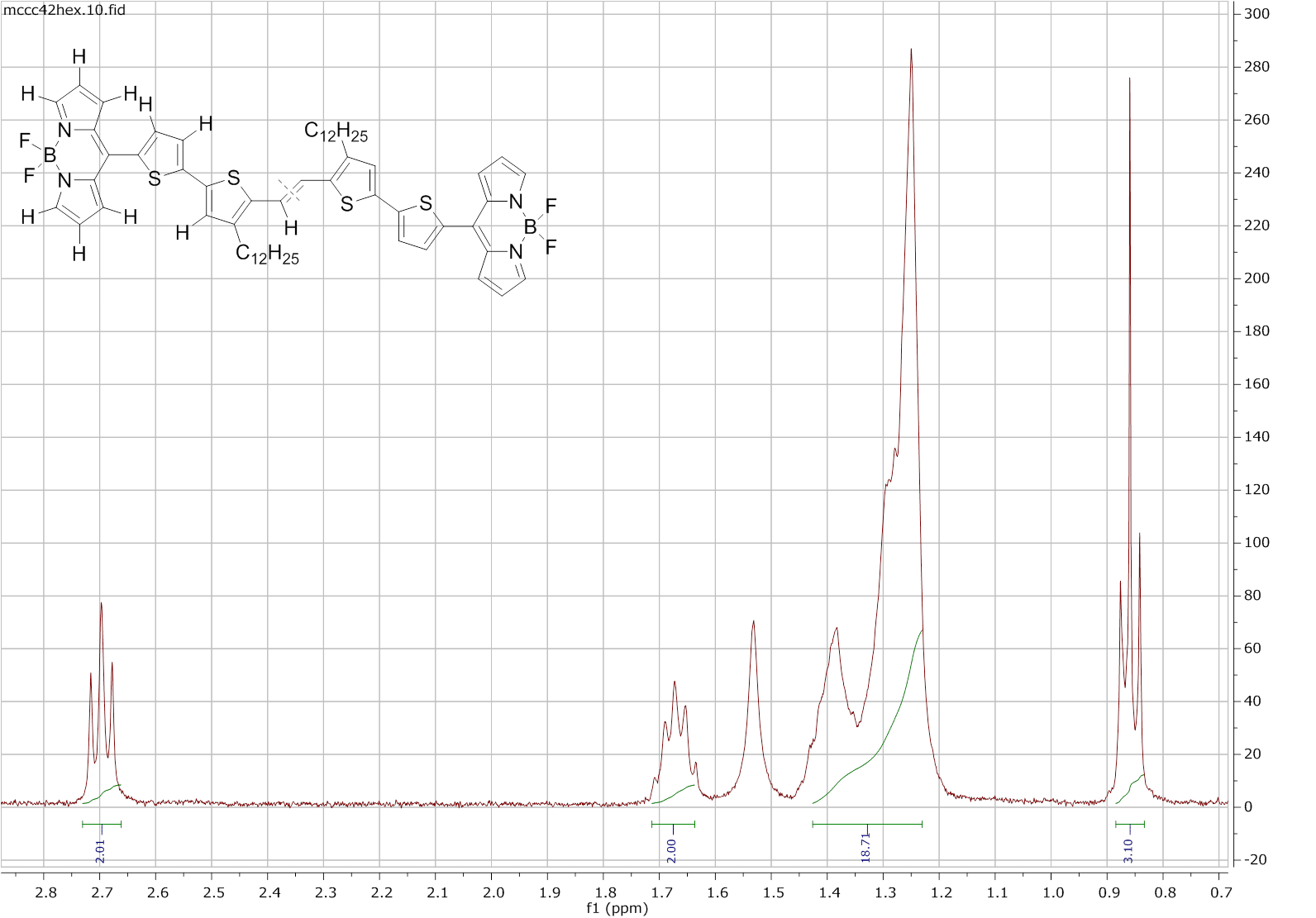
**

**Figure S4**. ^1^H-NMR of NIRBDTE (aliphatic region).

**MALDI-TOF Analysis of NIRBDTE**

NIRBDTE has been further analysed by MALDI-TOF (Bruker Reflex TOF in MPI Mainz) and the spectra is presented in Figure S5. As can be seen, one main peak at 1073,2539 which corresponds to the molecular weight of NIRBDTE is appeared.


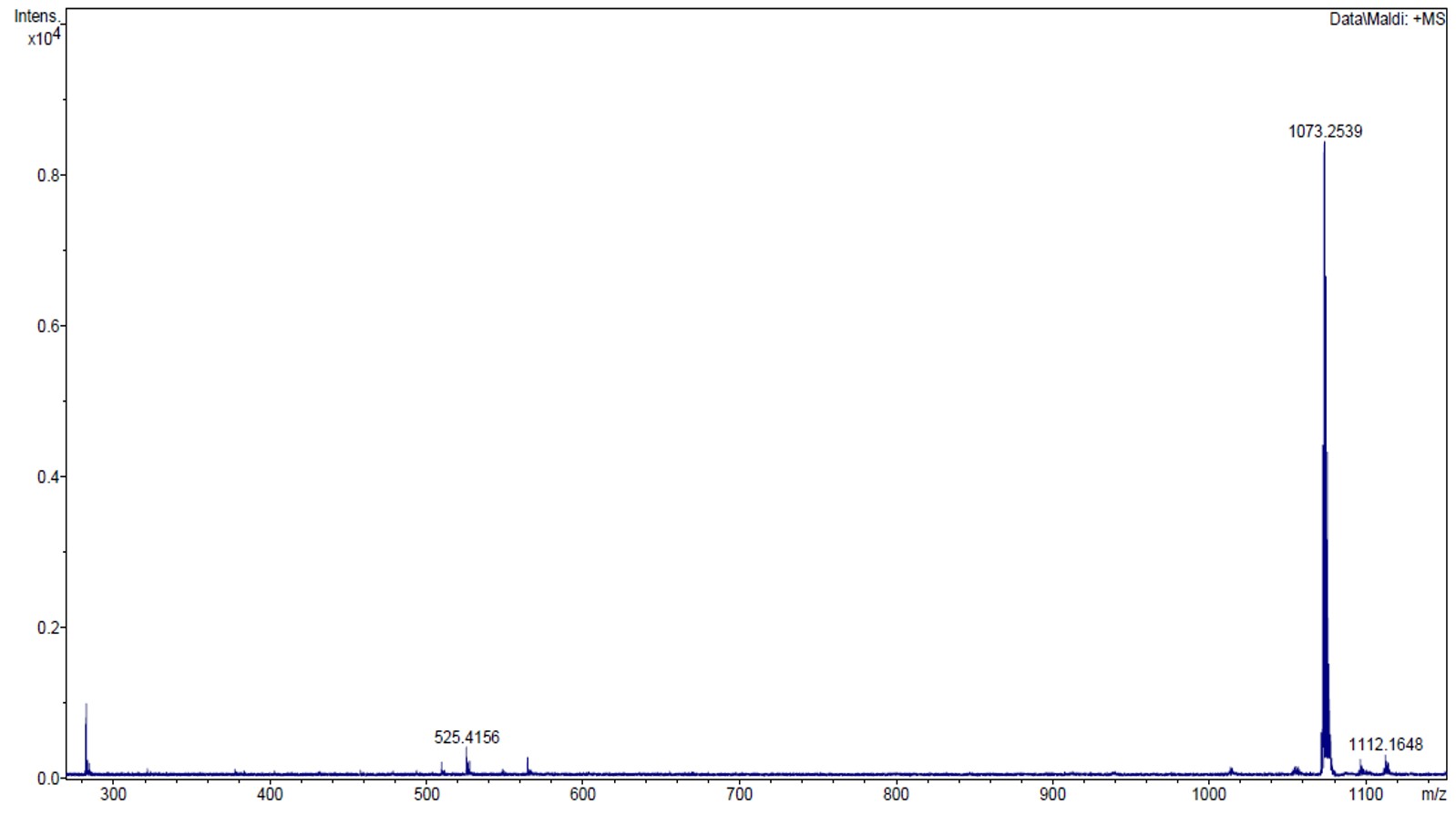


**Figure S5**. MALDI-TOF spectrum of NIRBDTE.

**Theoretical Calculations**

All calculations of the model compounds studied in this work have been performed using the Gaussian 03 software package.^[1]^ The ground-state geometry has been determined by a full geometry optimization of its structural parameters using the DFT, upon energy minimization of all possible isomers. In this work, the DFT calculations were performed using the B3LYP/6-31G(d,p) basis set. No symmetry constraints were imposed during the optimization process. The geometry optimization has been performed with a tight threshold that corresponds to root mean square (rms) residual forces smaller than 10^-5^ au for the optimal geometry. The visualization of the molecular orbitals has been performed using GaussView 5.0.

As shown in Figure S6a, DFT calculations indicate that the NIRBDTE molecule exhibits a “pseudo” 2D conformation with the appearance of parts of the NIRBDTE lying both in-plane and out-of-plane due to *cis*-geometric isomerism of the ethylene bond. However, additional contribution from the presence of the two dodecyl side groups which are aligned opposite in order to minimize the steric hindrance cannot be excluded in favouring this 2D conformation. The dihedral angles between the two carbon atoms of the ethylene bond along with the connected carbon and sulphur atoms of the two neighbouring thiophene rings are 124.2^o^ and 8.5^o^, respectively (Figure 6c). In both in-plane and out-of-plane directions the inter-ring torsional angles are between 9-13° which ensures an efficient *π*-orbital delocalization along the donor part (Figure 6b). On the other hand, inter-ring torsional angles of 43.7° were calculated between the boron-dipyrromethene and the meso-thiophene units (Figure S6b). This angle is smaller as compared to those of previously reported meso-aromatic substituted BODIPY small molecules, which is probably due to the lack of *α,β*-pyrrole substituents and sterically less-encumbered nature of the five-membered thiophene ring.^[2]^ Thus, our structural approach should offer a significant advantage to enhance charge-transport in the solid state when compared to the previously reported BODIPY-based semiconductors.^[3]^ Comparable donor-acceptor dihedral angles were reported between thiophene and naphthalenediimide (NDI)/perylenediimide (PDI) units in several high-performance n-channel semiconductors.^[4-6]^

*
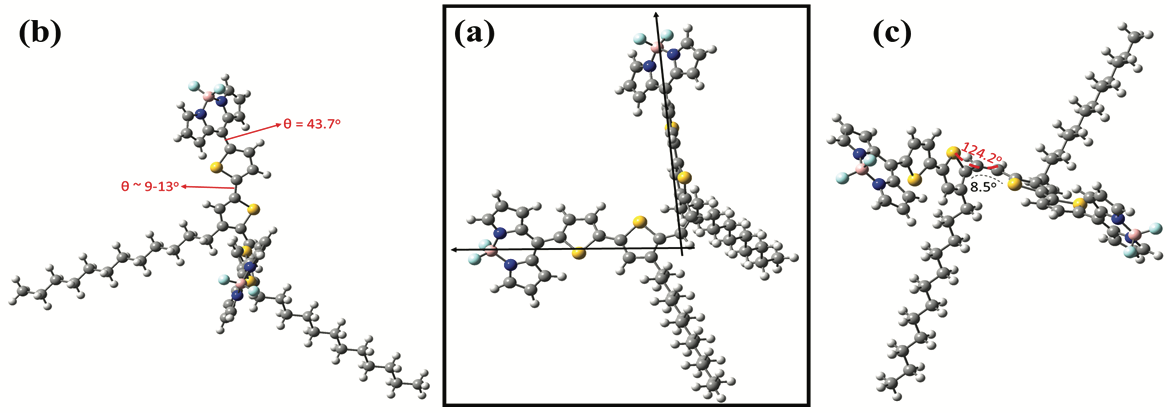
*

**Figure S6**. Optimized molecular geometries of NIRBDTE showing inter-ring torsional angles (θ) (DFT, B3LYP/6-31G(d,p).

**Atmospheric pressure photoelectron spectroscopy (AAPPS)**

AAPPS has been performed on a Riken Keiki AC-2 spectrometer in thin film at room temperature. The HOMO level as calculated by AAPPS (Figure S7) is situated at -5.48 eV in very good agreement with the theoretical predicted HOMO level (-5.56 eV).


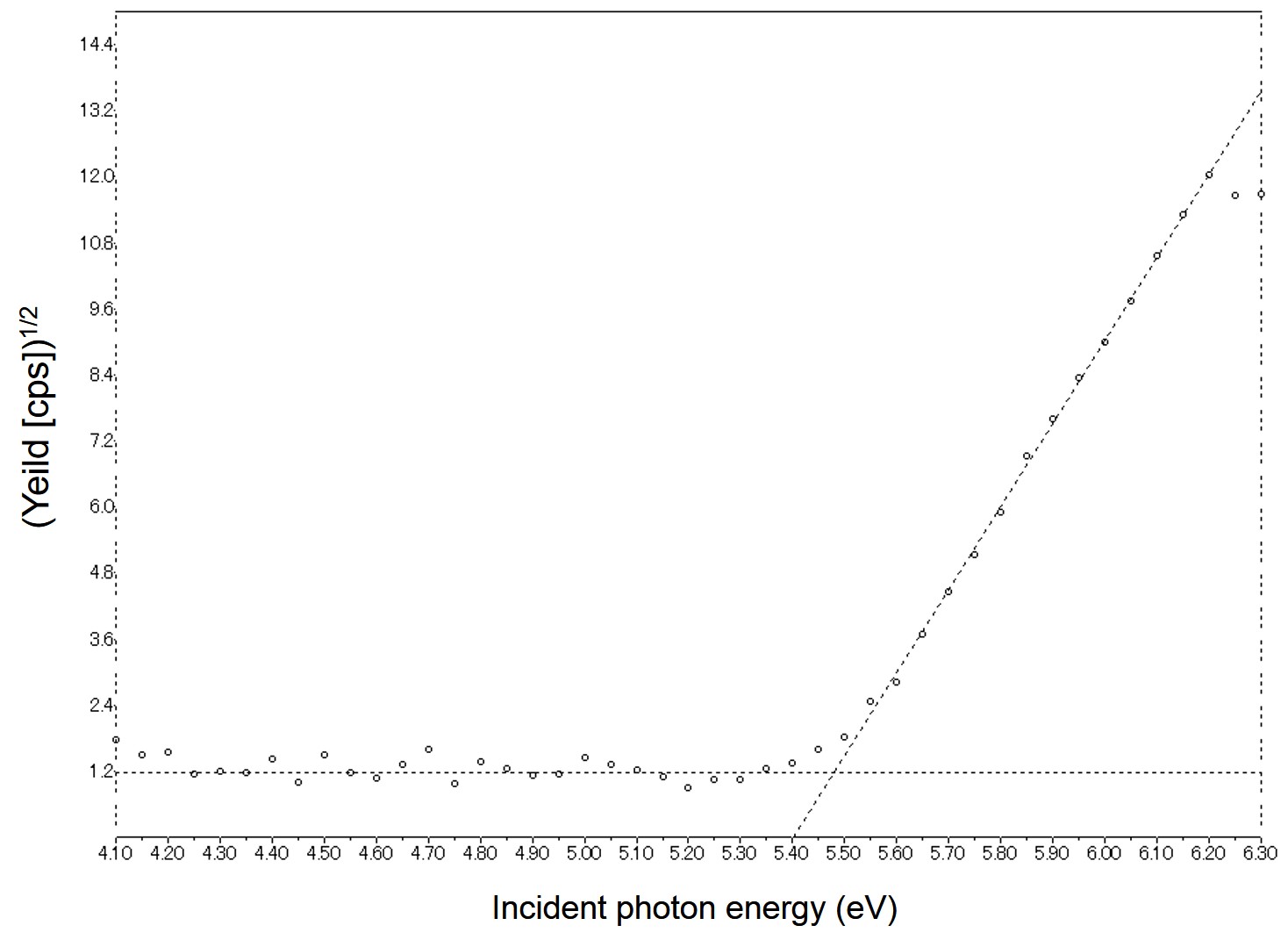


**Figure S7**. HOMO energy level of NIRBDTE estimated by atmospheric pressure photoelectron spectroscopy (AC-2).

**Cyclic voltammetry (CV) measurements**

CV studies were performed using a standard three-electrode cell. The oxidation and reduction potentials were calibrated against a ferrocene/ferrocenium (Fc/Fc^+^) redox couple, then they were referenced against saturated calomel electrode (SCE). Measurements were recorded using a PAR potensiostat/galvanostat Model VersaSTAT4, which was connected to a personal computer running VersaStudio software version 2.44. The cyclic voltammetry graphs were recorded at a potential scan rate of 100 mV s^-1^.

Working Solution: 10 mg of NIRBDTE / 0.1 M TBAP in CH_2_Cl_2_ (VWR, for HPLC, ≥ 99.9 %, distilled over P_2_O_5_) -5 mL-

Temperature: 25 °C

Atmosphere: dry argon (Ar) (bubbling 10 min and keeping over the sol.)

Working Electrode: Platinum (Pt) (diameter = 1 mm)

Counter Electrode: Pt wire

Reference Electrode: Pseudoreference silver (Ag°) wire


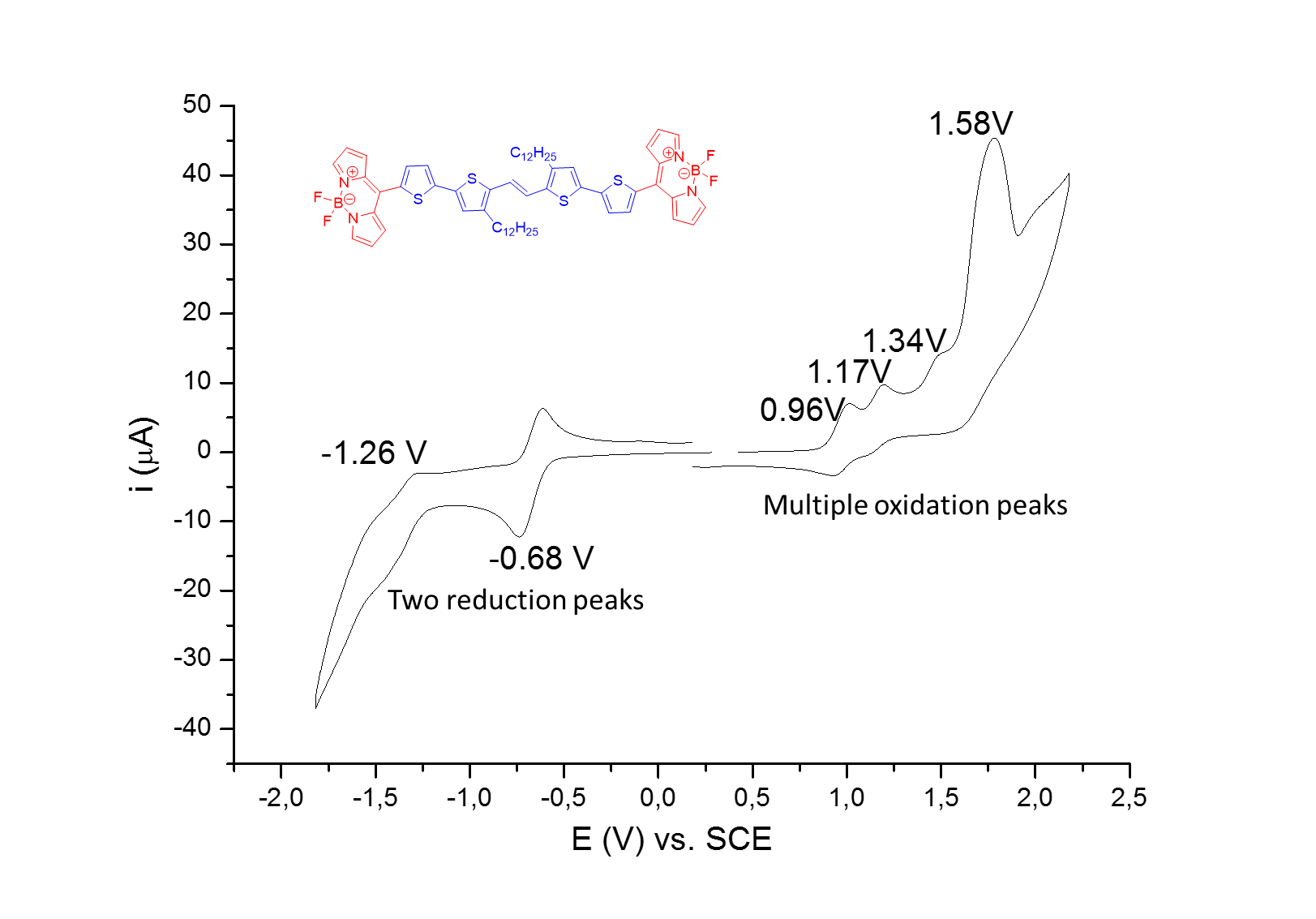


**Figure S8**. Cyclic voltammetry analyses of NIRBDTE.

The HOMO and LUMO energy levels have been determined using the following equations:

E_HOMO_ = - (E_ox_,^onset^ vs SCE + 4.7) eV

E_LUMO_ = - (E_red_,^onset^ vs SCE + 4.7) eV

E_ox_^onset^ = 0.96 V vs SCE

E_red_^onset^ = -0.68 V vs SCE

E_HOMO_ = -5.66 eV (HOMO)

E_LUMO_ = -4.02 eV (LUMO)

E_g_^electrochemical^= 1.64 eV

For the peak at 1.17 V: E_HOMO-1_ = -(1.17 + 4.7) = -5.87 eV

For the peak at 1.34 V: E_HOMO-2_ = -(1.34 + 4.7) = -6.04 eV

For the peak at 1.58 V: E_HOMO-3_ = -(1.58 + 4.7) = -6.28 eV

For the peak at -1.26 V: E_LUMO+1_ = -(-1.26 + 4.7) = -3.44 eV

Having a closer look to the reduction potentials we note that the reduction at -0.68 V is clearly reversible which is typical of BODIPYs.^[7]^ Therefore, if we assign this reduction process to the BODIPY and taking into account that the electrochemical band gap of BODIPY is 2.26 eV^[8]^ then the oxidation process at 1.58 V can also be assigned to BODIPY units. Furthermore, the other three distinct oxidation processes should be attributed to the oligothiophene central segment.

In summary, we see that NIRBDTE shows two reversible reduction peaks and four reversible oxidation peaks. The reversible oxidation and reduction peaks are calculated by [(Ep,c+Ep,a)/2] where Ep,c and Ep,a are the cathodic and anodic peak potentials, respectively. The reduction peaks at -0.68 V and -1.26 V versus saturated calomel electrode (SCE) are attributed to E_LUMO_ (-4.02 eV) and E_LUMO+1_ (-3.44 eV), respectively (Figure 1b). In addition, the oxidation peaks at 0.96 V, 1.17 V, 1.34 V and 1.58 V versus SCE are attributed to E_HOMO_ (-5.66 eV), E_HOMO-1_ (-5.87 eV), E_HOMO-2_ (‑6.04 eV) and EHOMO-3 (-6.28 eV), respectively (Figure 1b). From comparison of Figure S8 with typical voltammograms of BODIPY,^[7]^ we can safely assign the reduction peak at -0.68V and the oxidation peak at 1.58 V to the BODIPY units of the NIRBDTE, hence the reduction peak at ‑1.26 V and the oxidation peaks at 0.96 V, 1.17 V and 1.34 V can be assigned to the oligothiophene central segment.

**Time-resolved PL measurements**


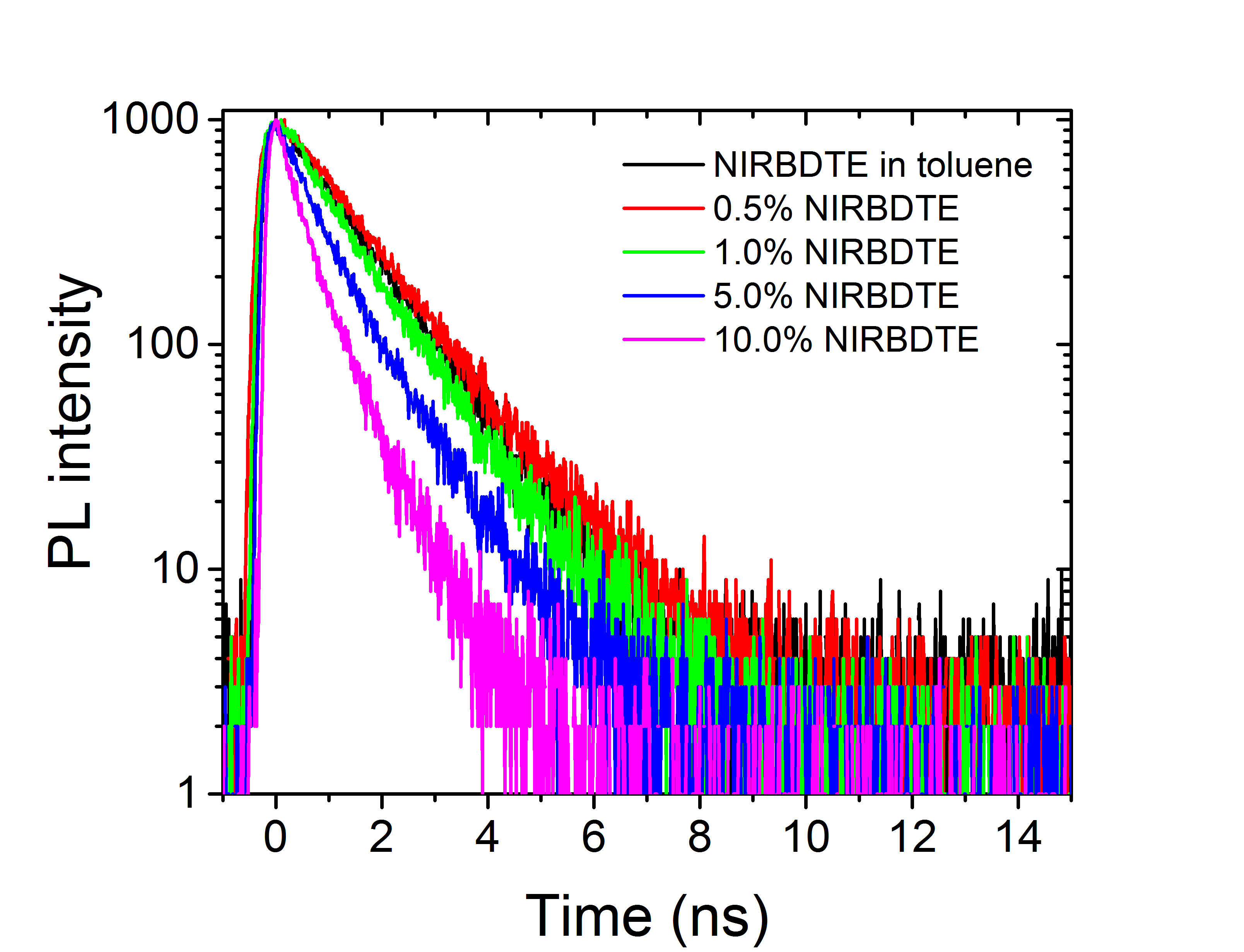


**Figure S9**. PL decay measured at 720 nm for the F8BT/oligomer blends and oligomer in toluene solution. PL decays were measured via time-correlated single-photon counting (TCSPC) technique with a 375 nm picosecond pulsed exciting laser. The decay for all blends is single exponential and varies from 1.2 ns (toluene solution) to 0.6 ns for the blend with 10% oligomer concentration.

**Type I and II Heterojunctions**


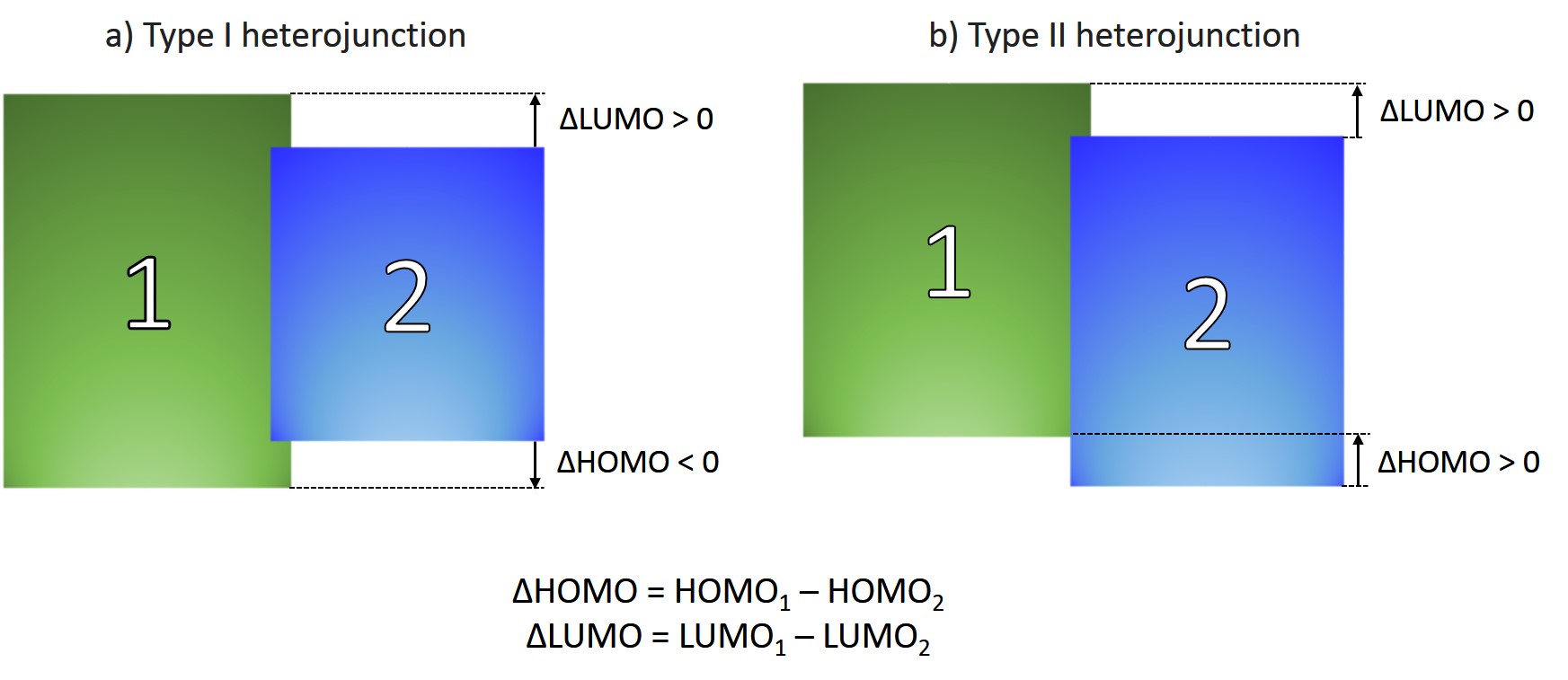


**Fig. S10** – Scheme of type I (a) and type II (b) heterojunctions.

**AFM images**

The images have been acquired with JPK Nanowizard 3 ULTRA speed AFM.


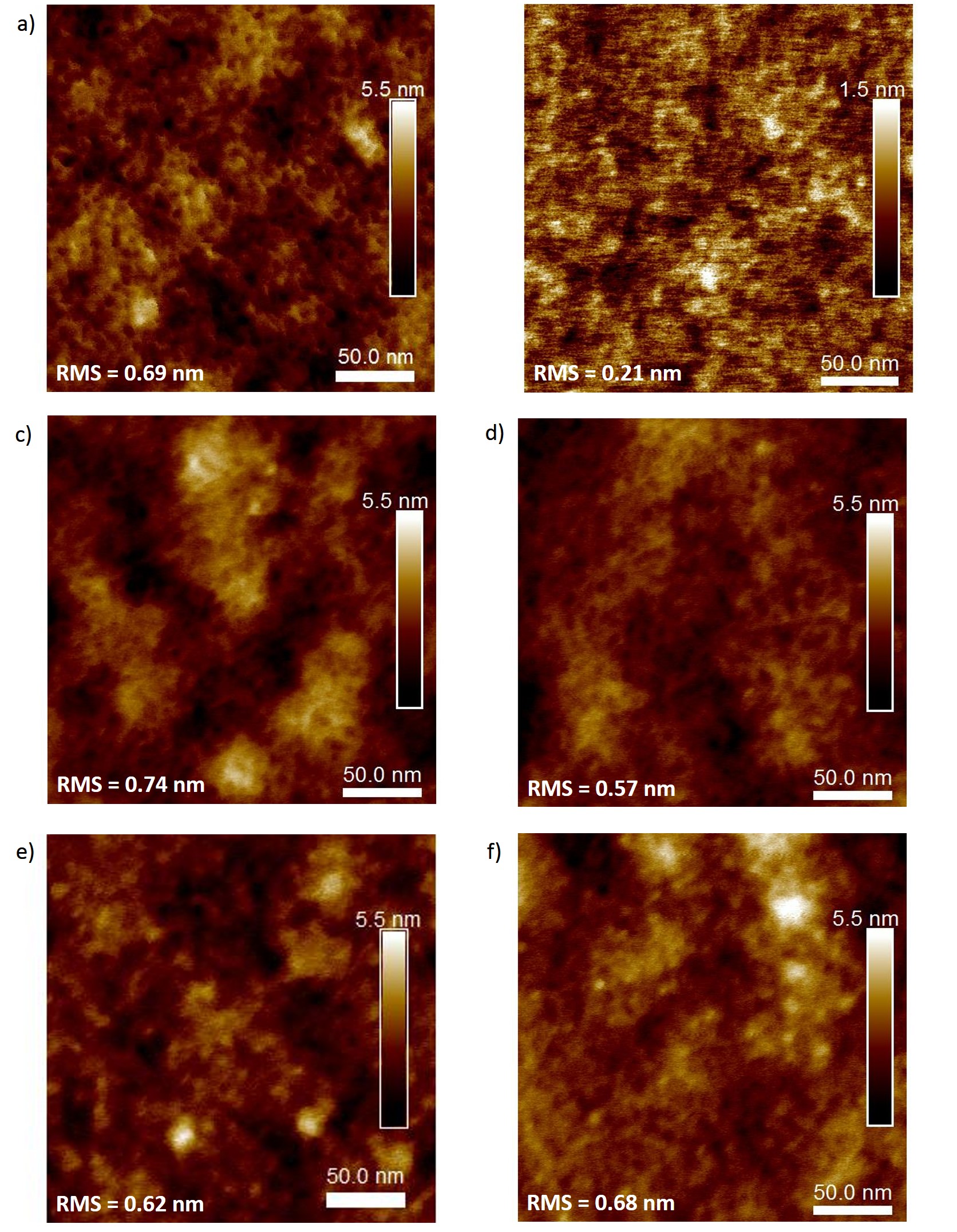


**Figure S11** – Peak force tapping AFM images of both neat F8BT (a) and NIRBDTE (b) and F8BT incorporating (c) 0.5%, (d) 1%, (e) 5% and (f) 10%wt NIRBDTE. The root-mean-square (RMS) roughness of each spin-cast film is reported in the related figure.

**PLEDs characteristics**


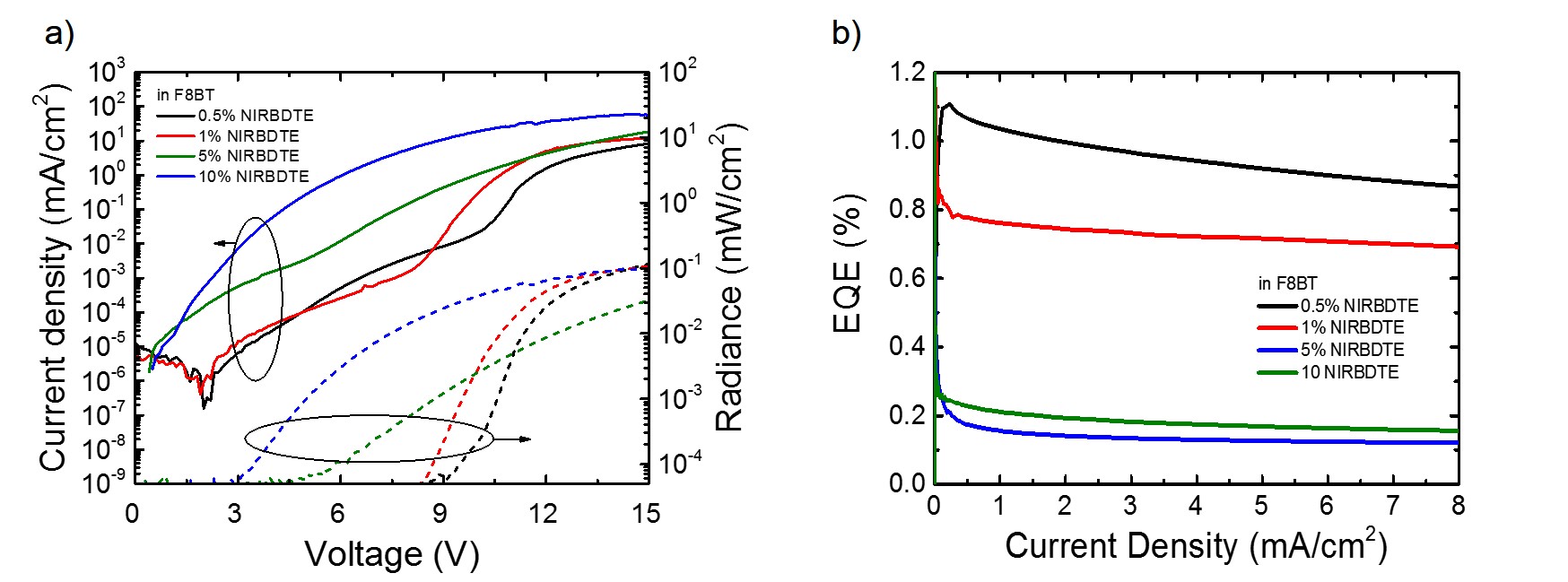


**Figure S12**. (a) Current density (solid line) and radiance versus voltage (dashed line) and (b) EQE versus current density curves of PLEDs incorporating 0.5% (black line), 1% (red line), 5% (green line) and 10% NIRDBTE loadings (red line) in F8BT. Interestingly we notice a clear correspondence between V_ON_ in the radiance vs. voltage curves, and the presence of a well-defined change of slope in the current density vs. voltage plots (for concentrations less than 10%wt), as expected when passing from unipolar to bipolar current transport (electrons are injected first in the F8BT matrix, owing to the low-lying LUMO of F8BT and its generally good electron transport properties).

**REFERENCES**

[1] Gaussian 03, Revision E.01,

M. J. Frisch, G. W. Trucks, H. B. Schlegel, G. E. Scuseria, M. A. Robb, J. R. Cheeseman, J. A. Montgomery, Jr., T. Vreven, K. N. Kudin, J. C. Burant, J. M. Millam, S. S. Iyengar, J. Tomasi, V. Barone, B. Mennucci, M. Cossi, G. Scalmani, N. Rega, G. A. Petersson, H. Nakatsuji, M. Hada, M. Ehara, K. Toyota, R. Fukuda, J. Hasegawa, M. Ishida, T. Nakajima, Y. Honda, O. Kitao, H. Nakai, M. Klene, X. Li, J. E. Knox, H. P. Hratchian, J. B. Cross, V. Bakken, C. Adamo, J. Jaramillo, R. Gomperts, R. E. Stratmann, O. Yazyev, A. J. Austin, R. Cammi, C. Pomelli, J. W. Ochterski, P. Y. Ayala, K. Morokuma, G. A. Voth, P. Salvador, J. J. Dannenberg, V. G. Zakrzewski, S. Dapprich, A. D. Daniels, M. C. Strain, O. Farkas, D. K. Malick, A. D. Rabuck, K. Raghavachari, J. B. Foresman, J. V. Ortiz, Q. Cui, A. G. Baboul, S. Clifford, J. Cioslowski, B. B. Stefanov, G. Liu, A. Liashenko, P. Piskorz, I. Komaromi, R. L. Martin, D. J. Fox, T. Keith, M. A. Al-Laham, C. Y. Peng, A. Nanayakkara, M. Challacombe, P. M. W. Gill, B. Johnson, W. Chen, M. W. Wong, C. Gonzalez, and J. A. Pople,

Gaussian, Inc., Wallingford CT, 2004.

[2] Yang, Y.; Guo, Q.; Chen, H.; Zhou, Z.; Guo, Z.; Shen, Z. Chem. Commun. 2013, 49, 3940-3942.

[3] Fabiano, S.; Usta, H.; Forchheimer, R.; Crispin, X.; Facchetti, A.; Berggren, M. Adv. Mater. 2014, 26, 7438-7443.

[4] Choi, J.; Kim, K.-H.; Yu, H.; Lee, C.; Kang, H.; Song, I.; Kim, Y.; Oh, J. H.; Kim, B. J. Chem. Mater. 2015, 27, 5230-5237.

[5] Usta, H.; Newman, C.; Chen, Z.; Facchetti, A. Adv. Mater. 2012, 24, 3678-3684.

[6] Chen, Z.; Zheng, Y.; Yan, H.; Facchetti, A. J. Am. Chem. Soc. 2009, 131, 8-9.

[7] a) A. B. Nepomnyashchii, M. Bröring, J. Ahrens, A. J. Bard J. Am. Chem. Soc. 2011, 133, 8633–8645; b) S. Rihn, M. Erdem, A. De Nicola, P. Retailleau, R. Ziessel Org. Lett. 2011, 13, 1916-1919.

[8] Economopoulos, S. P.; Chochos, C. L.; Ioannidou, H. A.; Neophytou, M.; Charilaou, C.; Zissimou, G. A.; Frost, J. M.; Sachetan, T.; Shahid, M.; Nelson, J.; Heeney, M.; Bradley, D. D. C.; Itskos, G.; Koutentis, P. A.; Choulis, S. A. *RSC Adv.* **2013**, *3*, 10221-10229
